# Editorial: Sleep and neurodegeneration

**DOI:** 10.3389/frsle.2025.1616848

**Published:** 2025-06-03

**Authors:** Robert T. R. Huckstepp, Chenjuan Gu

**Affiliations:** ^1^School of Life Sciences, University of Warwick, Coventry, United Kingdom; ^2^Research & Development, GlaxoSmithKline, Shanghai, China

**Keywords:** sleep, sleep apnoea, cognitive impairment, stroke, hypertension, amyloid burden affective disorders

Sleep is not just a time to take a temporary respite from the world and dream of a brighter tomorrow. While considered a time of replenishment and repair, it is also a period of growth and development, where memories are consolidated, and our immune systems bolstered. Of all the functions attributed to sleep, none are more important than those that occur in the brain; removal of waste products protect the brain from damage and allow the brain to function optimally. Sleep abnormalities, such as insomnia, and Irregular Sleep-Wake Rhythm Disorder (ISWRD) and sleep disordered breathing, are associated with Alzheimer's disease (Ungvari et al., 2025; Videnovic and Cai, 2025), vascular dementia (Ungvari et al., 2025), Huntington's disease (Voysey et al., 2025), the alpha synucleionopathies (Pérez-Carbonell and Iranzo, 2025), as well as lesser forms of cognitive decline such as mild cognitive impairment (Foukarakis et al., 2025). Even a single night's disrupted sleep can lead to important molecular changes, which are associated with increases in microRNAs that connect to neurodegenerative disease (Zhang et al., 2025). Sleep architecture can predict cognitive flexibility in Alzheimer's disease patients and correlates well with known biomarkers (Páez et al., 2025), and can predict phenoconversion in Parkinson's disease patients (Stefani et al., 2025). Altered REM sleep not only predicts alpha synucleionopathies, but its onset can even determine which brain regions have been affected and the pattern of spread of neurodegeneration (Schröter et al., 2025; Wang et al., 2025). Whilst research has often focused on how neurodegenerative disease either cause sleep disturbances or are exacerbated by them, more recently it has become apparent that regardless of which comes first, the relationship between sleep and neurodegeneration is bidirectional (Wang and Holtzman, 2020; Voysey et al., 2021; Antelmi et al., 2025). This Research Topic, therefore, aims to understand what happens when this relationship goes awry, and how impairments of sleep lead to neurodegenerative disease.

Ischemia stroke is a major neurological disease world-wide, and is a major cause of cognitive impairment (Elendu et al., 2023). Understanding that the neurological loss in stroke induces alterations in sleep, which in turn exacerbate neurodegeneration, in this topic, Fu et al., investigated hypocretin-1 as biomarkers of poor sleep quality in stroke patients. Low levels of hypocretin-1 are a predictor of tissue damage spread following stroke (Kotan et al., 2013): Could this be related to its effect on poor sleep quality or how it interplays with blood pressure and cerebral blood flow, as posited by Fu et al., and could it later determine the cognitive impairment seen in post-stroke patients?

Lv et al., looked to understand the association between poor sleep quality and cognitive impairment in elderly hypertensive patients. Both hypertension and sleep disturbances are linked to vascular dementia and cognitive impairment (Elwood et al., 2011; Emdin et al., 2016), suggesting a convergent pathway. The association between poor sleep quality and cognitive impairment in hypertensive patients spanned multiple educational levels, and affected a broad array of cognitive areas. Lv et al. further suggested that this relationship may be due to the deposition of brain amyloid β-protein (Aβ).

Kollarik et al. have shown that enhanced slow-wave activity induced by sodium oxybate (SO) lowered amyloid burden and improved cognitive performance in early stages of Alzheimer's disease. SO, a common treatment for narcolepsy, improved cognitive performance and lowered amyloid burden through increasing delta activity gain. Decreased delta power such as that associated with Alzheimer's disease, can be caused by disorders such as obstructive sleep apnoea (Monegro et al., 2019).

Hong et al. explored the effects of sleep apnoea on cognitive impairment and concomitant affective disorders. Whilst sleep apnoea has long been linked to the progression and severity of affective disorders (Kim et al., 2019), and also with cognitive decline (Marchi et al., 2024), Hong et al. investigate how these comorbidities interact with one another. Interestingly, it is the interplay between obstructive sleep apnoea and anxiety that is most strongly associated with cognitive impairment.

Jones et al. investigated the environmental and contextual factors that underlie cognitive impairment in sleep deprived patients. Whilst sleep is well known to cause cognitive impairment (Keil et al., 2023), which cognitive facets short-term sleep deprivation effects is less well understood. After extracting brain vital signs from established event-related potential components, basic attention was impaired by sleep deprivation, meaning that it is possible to use such vital signs to identify situation cognitive impairment related to acute sleep deprivation.

Given the infancy of how sleep and neurodegeneration bidirectionally interact, and the relevance of studies from seemingly disparate cognitive disorders, it will be interesting to see if sleep disturbances have broader effects that interact with multiple neurodegenerative disorders, whether it's interactions with each disorder are more specific, or if it is a compilation of both.
